# Impact of Ligamentous Adhesion to the Posterior Cruciate Ligament on Radiological, Arthroscopic, and Clinical Outcomes One Year After ACL Reconstruction: A Cohort Study -

**DOI:** 10.31661/gmj.v14i.3589

**Published:** 2025-12-29

**Authors:** Ali Yeganeh, Shayan Amiri, Mehdi Moghtadaei, Pedram Doulabi, Javad KhajeMozafari, Ahmad Hemmatyar, Amir Mehrvar, Khatere Mokhtari, Mohammad Eslami Vaghar

**Affiliations:** ^1^ Trauma and Injury Research Center, Rasoul Akram Hospital, Department of Orthopaedic, School of Medicine, Iran University of Medical Sciences, Tehran, Iran; ^2^ Shohadaye Haftom-e-tir Hospital, Department of Orthopaedic, School of Medicine, Iran University of Medical Sciences, Tehran, Iran; ^3^ Department of Orthopaedic, School of Medicine, Iran University of Medical Sciences, Tehran, Iran; ^4^ School of Medicine, Shahroud University of Medical Sciences, Shahroud, Iran; ^5^ Department of Orthopedics, Taleghani Hospital Research Development Committee, Shahid Beheshti University of Medical Sciences, Tehran, Iran; ^6^ Department of Cell and Molecular Biology and Microbiology, Faculty of Biological Science and Technology, University of Isfahan, Isfahan, Iran; ^7^ Department of Gynecology, Faculty of Medicine, Tehran Medical Sciences, Islamic Azad University, Tehran, Iran; ^8^ Farhikhtegan Medical Convergence Sciences Research Center, Farhikhtegan Hospital Tehran Medical Sciences, Islamic Azad University, Tehran, Iran

**Keywords:** Anterior Cruciate Ligament, Posterior Cruciate Ligament, Ligament Adhesion, Meniscus Injuries, Cartilage Injuries

## Abstract

**Background:**

The anterior cruciate ligament (ACL) and posterior cruciate ligament (PCL) play a crucial role in maintaining knee stability by controlling anterior and posterior tibial translation. After ACL reconstruction, residual ACL tissue may adhere to the PCL, potentially altering knee biomechanics and affecting postoperative recovery. The clinical significance of this adhesion remains uncertain. This study aimed to evaluate the relationship between ACL–PCL adhesion and radiological, arthroscopic, and clinical outcomes one year after ACL reconstruction.

**Materials and Methods:**

This retrospective cohort study included patients with ACL tears who underwent reconstructive surgery at hospitals in Tehran between 2022 and 2023. Patients were divided into two groups based on arthroscopic findings: those with ACL remnant adhesion to the PCL and those without adhesion. Demographic data, postoperative MRI findings (chondral lesions, articular cartilage damage, meniscal injuries, varus deformity, and concomitant ligament injuries), and clinical outcomes assessed by Lachman and pivot shift tests were compared between groups. Statistical analysis was performed using SPSS software using chi-square and McNemar’s tests.

**Results:**

A total of 87 patients were evaluated (mean age 30.42 ± 5.79 years), including 78 males and 9 females. ACL remnant adhesion to the PCL was observed in 74 patients (85.1%). Articular cartilage damage was more frequent in the non-adhesion group (23.1%). Medial meniscal injuries were present in 56.3% of patients and were more common in the non-adhesion group (76.9%). Lateral and root meniscal injuries, as well as concomitant MCL and PCL injuries, were more frequently observed in the adhesion group. Varus deformity showed no significant association with adhesion status. No significant differences were found in Lachman or pivot shift test results, and adhesion was not associated with age or gender.

**Conclusion:**

ACL remnant adhesion to the PCL is a common finding after ACL reconstruction but was not associated with adverse radiological findings or short-term clinical outcomes. Further studies are needed to assess its long-term clinical relevance.

## Introduction

The posterior cruciate ligament (PCL) and anterior cruciate ligament (ACL) are essential components of the knee joint's complex stabilizing system, playing complementary roles in maintaining its biomechanical integrity. The ACL primarily prevents anterior translation of the tibia relative to the femur, safeguarding against excessive forward movement that could compromise joint stability. Conversely, the PCL is responsible for resisting posterior translation of the tibia, countering forces that might push the tibia backward. Together, these ligaments ensure the knee remains functional during various dynamic activities, such as walking, running, and pivoting. Disruption or injury to either ligament, particularly in the context of trauma or overuse, can significantly impair knee stability and functionality, highlighting their critical roles in both joint movement and overall structural equilibrium [1][2][3][4].

Understanding the intricate relationship between the ACL and PCL is vital for addressing injuries and optimizing surgical and rehabilitative strategies.

Approximately half of ACL injuries are associated with damage to other parts of the knee, such as joint cartilage, menisci, and other ligaments. Specifically, 50% of ACL injuries involve meniscal damage, 30% involve cartilage damage, and another 30% are associated with collateral ligament injuries [5]. The global incidence of ACL injuries is estimated to be between 100,000 and 250,000 cases annually [5].

The primary goal of ACL reconstruction surgery is to restore knee stability. To achieve this and minimize the complications associated with tendon graft harvesting, various surgical techniques have been developed. Many orthopedic surgeons consider magnetic resonance imaging (MRI) a valuable tool for diagnosing intra-articular knee injuries, especially when clinical examinations are inconclusive [6]. In an injured ligament, cell-cell adhesion and the extracellular matrix play critical roles in physiological processes like wound healing, immune regulation, thrombosis, and hemostasis [7][8]. In injured ligaments, fibroblasts within the disordered tissue matrix migrate to the injury sites. This migration, essential for wound closure during the healing process, involves adhesion processes and the reorganization of the internal cytoskeleton through the remodeling of actin filaments and other cytoskeletal proteins. Recent research underscores the crucial role of cytoskeletal proteins in cell adhesion and motility [9].

This adhesion can pose considerable challenges for the patient during the healing process. Several intrinsic and extrinsic risk factors are linked to ACL injuries, which can be classified into modifiable and non-modifiable categories. Non-modifiable intrinsic factors include gender, anatomical variations, previous ACL injuries, and genetic predisposition, while modifiable intrinsic factors include body mass index (BMI), hormonal status during sports participation, neuromuscular deficits, and biomechanical abnormalities [10].

ACL reconstruction surgery is a widely utilized procedure aimed at restoring knee stability following an ACL injury [11]. Several factors can significantly influence the long-term outcomes of ACL reconstruction surgery, with ligamentous adhesion to the posterior cruciate ligament (PCL) being one of the most critical. These adhesions may result in restricted knee movement, chronic pain, and recurrent instability.

Consequently, investigating the relationship between PCL adhesions and postoperative radiological, arthroscopic, and clinical findings could offer valuable insights for improving treatment strategies and preventing complications. Despite the high frequency of these injuries, accurate data on their prevalence in Iran remains limited.

The novelty of this study lies in its focus on ACL remnant to PCL adhesions, an underexplored area in knee surgery research, particularly in the Iranian population, where data is scarce. This research will provide new insights into the role of PCL adhesions in long-term knee complications and guide improvements in postoperative management and treatment strategies.

It aimed to This study aims to evaluate the correlation between ligamentous adhesion to the PCL and radiological, arthroscopic, and clinical findings one-year post-surgery in patients with ACL tears.

## Materials and Methods

### Study Setting and Time

This retrospective cohort study analyzed data from patients with ACL tears who were treated with ACL reconstruction surgery at Moheb Mehr and Rasoul Akram Hospitals (Tehran, Iran) between 2022 and 2023. Given that the patients' surgeries and postoperative follow-up had already been completed, we employed a retrospective cohort design to investigate the relationship between ACL remnant adhesion to the PCL and various clinical, radiological, and arthroscopic outcomes one year post-surgery.

These institutions are well-established centers for orthopedic surgeries, including ACL reconstruction procedures. The study included 87 patients, with 74 in the ACL remnant adhesion group and 13 in the non-adhesion group.

### Study Participants

The study consisted of patients diagnosed with cruciate ligament injuries, specifically anterior cruciate ligament (ACL) tears associated with posterior cruciate ligament (PCL) adhesions, who underwent surgical reconstruction. patients with ACL tears undertaking reconstructive surgery. Exclusion criteria: none.

### Study Arms

Participants were divided into two groups based on intraoperative arthroscopic findings: ACL remnant adhesion group and non-adhesion group.

### Study Variables/Definitions

The study variables were classified as follows: 1) Background Features: Age (in years) and sex (female/male); 2) Radiological Features: Including chondral lesions, articular cartilage damage, meniscal injuries (medial, lateral, and root injuries), and varus deformity. The presence or absence of these features was recorded as categorical data, and statistical comparisons were made using the chi-square test; 3) Arthroscopic Features: Including the findings from the Lachman and pivot shift tests, which were used to evaluate ligament stability.

These tests were recorded as positive or negative for each patient and analyzed through chi-square comparison; 4) Clinical Features: The results of the Lachman and pivot shift tests were included, assessing clinical instability and its relationship with ACL remnant adhesion status.

### Clinical Outcomes

Evaluated using physical examination findings, such as Lachman and pivot shift tests. Radiological outcomes: Postoperative magnetic resonance imaging (MRI) was performed one year after surgery to assess chondral lesions, articular cartilage damage, meniscal injuries, varus deformity, and injuries to other cruciate ligaments. Arthroscopic outcomes: Based on intraoperative findings related to ACL and PCL adhesions and associated injuries.

### Surgical Approach

All surgeries were conducted by two experienced surgeons using a standardized approach. Both surgeons utilized two portals: the anteromedial and anterolateral portals. The surgical technique involved either an allograft or hamstring graft procedure, with no use of patellar or patellar bone grafts. Femoral fixation was achieved using an Endobutton, while tibial fixation employed bioabsorbable screws.

The procedures were focused solely on ACL reconstruction; in cases where the PCL was partially torn, it was deemed not severe enough to require surgical intervention. If meniscal tears were present, they were managed intraoperatively with suturing or meniscal shaving. Chondral lesions up to grade 3 were treated with drilling or abrasive chondroplasty. All fixation devices (Femoral Fixation Device) and (PLLA Btcp Bioabsorbable Screws) were sourced from BIOTURN (Arshin Salamat Sepanta Co., Tehran/Iran).

### Ethical Statement

This study received approval from the Ethics Committee of the university under ethics code IR.IUMS.FMD.REC.1402.356. All procedures were carried out in accordance with ethical standards, and no invasive procedures beyond routine clinical practice were performed. Informed written consent was obtained from all participants prior to their inclusion in the study.

### Sample Size Consideration

all eligible patients who met the inclusion criteria were included in the study.

### Statistical Analysis

The data analysis was performed using SPSS version 24 (IBM Corp. Released 2013. IBM SPSS Statistics for Windows, Version 24. Armonk, NY: IBM Corp), developed by IBM, including mean ± standard deviation (Mean ± SD) for quantitative variables and frequency and percentage for categorical variables, were used to summarize the data. The chi-square test was employed to assess demographic and outcome differences between the two groups. The independent t-test was used to compare the mean age between the groups. McNemar's test was used to compare the success rates of ligament repair and associated injuries between groups. A P-value of <0.05 was considered statistically significant. Relative risk (RR) and 95% confidence intervals (CI) were calculated using the standard formula for RR, with confidence intervals determined using the Wald method.

## Results

**Figure-1:**
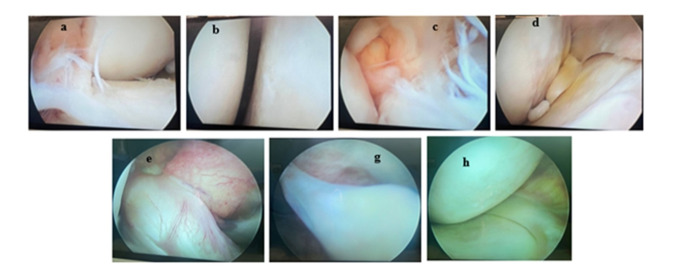
Figure 1. Various findings related to ACL remnant adhesion and associated knee injuries, categorized by specific types of lesions and adhesions. (A and C) ACL remnant not adhered to the PCL. (B) Type 2 condylar lesion on the tibial condyle. (D) Condylar lesion presenting as a lozenge-shaped defect. (E) ACL remnant fully adherent to the PCL. (G) Large tear of the medial meniscus. (H) Type 3 condylar lesion on the femoral condyle.

**Table:** Table1. Comparison of Mean Age and Gender Distribution Among All Patients and Study Groups

**Groups**	**Gender**	**Number (%)**	**P-value**
ACL remnant adhesion group (74)	Male	67 (90.2%)	
	Female	7 (9.8)	0.94
non-adhesion group (13)	Male	11 (90.9)	
	Female	2 (9.1)	
**Groups**	**Minimum-** **Maximum** **(Years old)**	**Mean ± (SD)**	**P-value**
ACL remnant adhesion group (74)	20-40	29.78 (5.48)	0.52
non-adhesion group (13)	18-40	32.82 (6.55)	

Data were analyzed using the chi-square test to evaluate gender distribution and the independent t-test for comparison of mean age between the groups. (ACL (Anterior Cruciate Ligament), PCL (Posterior Cruciate Ligament))

**Table:** Table2. Percentage Distribution of Chondral Lesions and Articular Cartilage Injuries Among All Patients

	**Groups**	**presence of Lesions**/ **Absence of Lesions**	**Number (%)**	**P-value**
Chondral Lesions	ACL remnant adhesion group (74)	Positive	1(1.4%)	
		Negative	73(98.6%)	
	non-adhesion group (13)	Positive	0	0.67
		Negative	13 (100%)	
Articular Cartilage Injuries	ACL remnant adhesion group (74)	Positive	15(20.3%)	
		Negative	59 (79.7%)	
	non-adhesion group (13)	Positive	3(23.1%)	0.81
		Negative	10 (76.9%)	

The chi-square test was used to assess the presence of chondral lesions and articular cartilage injuries between the two groups. (ACL (Anterior Cruciate Ligament), PCL (Posterior Cruciate Ligament))

**Table:** Table3. Percentage Distribution of Types of Meniscus Injuries Among All Patients

	**Groups**	**Presence of Injury / Absence of Injury **	**Number (%)**	***P*** **-value**
Medial Meniscus Injury	ACL remnant adhesion group (74)	Positive ** **	39(52.7%)	
		Negative ** **	35(47.3%)	
	non-adhesion group (13)	Positive ** **	10 (76.9%)	0.10
		Negative ** **	3 (23.1%)	
Lateral Meniscus Injury	ACL remnant adhesion group (74)	Positive ** **	5(6.8%) ** **	
		Negative ** **	69(93.2%) ** **	
	non-adhesion group (13)	Positive ** **	0 ** **	0.33
		Negative ** **	13 (100%) ** **	
Meniscal root Injury	ACL remnant adhesion group (74)	Positive ** **	2(2.7%)	
		Negative ** **	72 (97.3%)	
	non-adhesion group (13)	Positive ** **	0	0.54
		Negative ** **	13 (100%)	

The chi-square test was applied to determine the association between ACL remnant adhesions to the PCL and types of meniscus injuries. (ACL (Anterior Cruciate Ligament), PCL (Posterior Cruciate Ligament))

**Table:** Table4. Percentage Distribution of Concurrent Cruciate Ligament Injuries Among All Patients

	**Groups**	**With**	**Number (%)**	***P*** **-value**
	ACL remnant adhesion group (74)	MCL	2 (2.7%)	
		PCL	4(5.4%)	
Concurrent Injuries to Other Cruciate Ligaments		No Concurrent Injuries	68(91.9%)	
	non-adhesion group (13)	MCL ** **	0	0.79
		PCL ** **	1 (7.7%)	
		No Concurrent Injuries ** **	12 (92.3%)	

The chi-square test was used to compare the incidence of concurrent cruciate ligament injuries between the two groups. McNemar's test was employed to compare the incidence of concurrent cruciate ligament injuries between the two groups. (ACL (Anterior Cruciate Ligament), PCL (Posterior Cruciate Ligament))

**Table:** Table5. Percentage Distribution of Varus Deformity Among All Patients

**Groups**	**Varus**	**Number (%)**	***P*** **-value**
ACL remnant adhesion group (74)	+	9 (12.2%)	
	-	65(87.7%)	
non-adhesion group (13)	+ ** **	1 (7.7%)	0.64
	- ** **	12 (92.3%)	

The chi-square test was applied to evaluate the occurrence of varus deformity in patients with and without ACL remnant adhesions to the PCL. (ACL (Anterior Cruciate Ligament), PCL (Posterior Cruciate Ligament))

**Table:** Table6. Percentage Distribution of Lachman and Pivot Shift Test Results Among All Patients

	**Groups**	**The test results**	**Number (%)**	***P*** **-value**
	ACL remnant adhesion group (74)	Positive ** **	71(95.9%)	
Lachman Test		negative ** **	3(4.1%)	
	non-adhesion group (13)	Positive ** **	0	0.46
		negative ** **	13 (100%)	
	ACL remnant adhesion group (74)	Positive ** **	71(95.9%)	
Pivot Shift Test	negative ** **	3(4.1%)		
	non-adhesion group (13)	Positive ** **	0	0.46
		negative ** **	13 (100%)	

The chi-square test was used to assess the distribution of Lachman and Pivot Shift test results between the two groups. (ACL (Anterior Cruciate Ligament), PCL (Posterior Cruciate Ligament))

### 1. Demographic Variable Frequencies among Study Participants

### 1. 1. Age and Its Impact on ACL-PCL Adhesion

The average age of all patients in this study was 30.42 ± 5.79 years. The mean age of the group with ACL remnant adhesion to PCL was 29.78 ± 5.48 years, while the group without adhesions had an average age of 32.82 ± 6.5 years. The study found no statistically significant relationship between ACL remnant adhesion to PCL and age (P-value>0.05). This suggests that age does not have a significant impact on the likelihood or severity of adhesions between the ACL remnant and PCL (Table-1).

### 1. 2. Gender and Age Its Impact on ACL-PCL Adhesion

In this study, a total of 93 participants were included, comprising 90.3% males and 9.7% females. The highest proportion of both males and females was observed in the group with ACL adhesion to PCL, consisting of 90.2% males and 9.8% females. No statistically significant relationship was found between ACL remnant adhesion to PCL and gender (P-value>0.05). The results are displayed in Table-1.

### 1. 3. Percentage Distribution of ACL to PCL Adhesion Among All Study Patients

A total of 85.1% of the study participants exhibited ACL remnant adhesion to PCL. This high prevalence suggests a significant occurrence of this condition among the patient population studied. The results, indicate that a substantial majority of patients had adhesions, highlighting the importance of addressing this issue in clinical settings.

### 1. 4. Incidence of Chondral Lesions and Articular Cartilage Injuries

In the study, 1.1% of all patients had chondral lesions, and 20.7% had articular cartilage injuries. The highest incidence of chondral lesions was observed in the group with ACL remnant adhesion to PCL (1.4%), while the highest percentage of articular cartilage injuries was found in the group without adhesions (23.1%). Although the number of individuals with cartilage injuries was higher in the adhesion group (15 individuals), there was no statistically significant relationship between ACL adhesion to PCL and the presence of chondral lesions or articular cartilage injuries (P-value>0.05) (articular cartilage injuries, RR=0.878, 95% CI: 0.47-1.66). The results are summarized in Table-2. For Chondral Lesions, it was not possible to calculate the Relative Risk (RR) and 95% Confidence Interval (CI) due to the absence of lesions in all patients of the non-adhesion group (Group 2). Since there were no cases of chondral lesions in the non-adhesion group, the calculation of RR and CI would not be meaningful. Instead, Fisher's Exact Test was applied to determine the statistical significance of the difference between the two groups. The results of Fisher's Exact Test yielded a P-value of 1.0, indicating that there was no statistically significant difference between the ACL remnant adhesion group (Group 1) and the non-adhesion group (Group 2) with respect to the presence of chondral lesions.

This suggests that the occurrence of chondral lesions in these two groups is not significantly different.

### 1. 5. Relationship Between ACL Adhesion to PCL and Meniscus Injuries

49 participants (56.3%) had medial meniscus injury, 5 participants (5.7%) had lateral meniscus injury, and 2 participants (2.3%) had Meniscal root Injury.

The highest incidence of medial meniscus injury was observed in the group without adhesion, at 76.9%. The highest incidence of lateral meniscus injury was in the group with adhesion, at 6.8%, and the highest incidence of root meniscus injury was also in the adhesion group, at 2.7%. There was no statistically significant relationship between ACL remnant adhesion to PCL and meniscus injury (P-value>0.05) (RR=0.685, 95% CI: 0.46-1.01). These findings underscore the complex nature of knee joint injuries and the need for further investigation into their underlying mechanisms (Table-3 and Figure-1).

### 1. 6. Relationship Between ACL Adhesion to PCL and Concurrent Ligament Injuries

In this study, 2.3% of participants had concurrent MCL injuries, and 5 participants (5.7%) had concurrent PCL injuries. The highest number of concurrent MCL injuries was observed in the adhesion group, with 2 participants (2.7%), while the highest number of concurrent PCL injuries again occurred in the adhesion group, with 4 participants (5.4%). There was no statistically significant relationship between ACL remnant adhesion to PCL and concurrent injuries to other cruciate ligaments (P-value>0.05) (PCL: RR = 0.541, 95% CI: 0.14-2.12). These findings suggest that while ACL remnant adhesion to PCL may influence specific ligament injuries, it does not significantly impact the overall occurrence of concurrent ligament injuries in the knee joint. The results are summarized in Table-4.

### 1. 7. Impact of ACL Adhesion to PCL on Varus Deformity

In this study, 11.5% of participants exhibited varus deformity, with the highest prevalence (12.2%) observed in the group with ACL remnant adhesion to PCL. However, statistical analysis did not reveal a significant association between ACL adhesion to PCL and the presence of varus deformity (P-value>0.05) (RR=1.585, 95% CI: 0.62-3.99). These findings suggest that while ACL remnant adhesion to PCL may coincide with varus deformity in a subset of cases, it does not appear to be a determining factor in its occurrence based on our study results (Table-5).

### 1. 8. Relationship Between ACL Remnant Adhesion to PCL and Clinical Examination Tests

In this study, the Lachman test showed a negative result in 71.4% of participants, with the highest prevalence (33.2%) observed in the group with ACL remnant adhesion to PCL. Similarly, the Pivot shift test yielded a negative result in 28.6% of participants, with the highest prevalence (66.8%) also seen in the adhesion group. However, statistical analysis did not find a significant association between ACL remnant adhesion to PCL and the results of these clinical examination tests (P-value>0.05). These findings suggest that while ACL remnant adhesion to PCL may coincide with certain clinical findings such as the Lachman and Pivot shift tests, it does not significantly influence their outcomes based on our study results. The findings are encapsulated in Table-6.

## Discussion

In our analysis, we focused on associated injuries, as they may worsen the effects of abnormal ligament adhesion. For example, cartilage damage could increase joint instability, and meniscal tears might further impair knee function over time. Ligamentous injuries combined with adhesions could complicate recovery and heighten the risk of post-surgical complications.

By exploring these correlations, our study aims to provide valuable insights into ACL injury management and the prevention of long-term joint damage. Additionally, a study evaluating 42 reconstructed ACLs using MRI found that impingement on the PCLs was present in both single-bundle and double-bundle reconstructions. At 3 months post-surgery, 14 of 31 single-bundle and 5 of 11 double-bundle reconstructions showed impingement, increasing to 17 and 5 cases, respectively, at 12 months. The PCL index was significantly lower in the impingement-positive group, indicating that ACL reconstructions with impingement exert greater posterior pressure on the PCL compared to the impingement-negative group [12].

In a study, it was reported that managing multiple-ligament-injured knees, often involving ACL, PCL, and collateral ligament tears, requires thorough vascular assessment and a systematic approach to diagnosis and treatment. Arthroscopically assisted ACL/PCL reconstruction was shown to improve postoperative stability. Additionally, MCL tears may be treated with bracing, while posterolateral corner injuries are best managed with primary repair and reconstruction using robust autografts or allografts. Surgical timing depends on the extent of ligament injury, vascular status, reduction stability, and the patient’s overall health. Allografts are preferred due to their strength and the absence of donor-site morbidity [13].

A similar study by I.K. Lo et al. examined the reattachment of torn ACLs in 101 patients undergoing ACL reconstruction with arthroscopy. The results showed that about 72% of these unstable knees had reattachment of the torn ACL to the PCL, while 18% showed no reattachment, and only 2% had completely absent ACLs. These findings suggest that complete resorption of the ACL is rare, even in chronic, functionally unstable knees. The study also indicates that torn ACLs generally reattach to the PCL through scar formation. Although the functional effectiveness of these reattachments is limited by factors such as reattachment location, quantity, and quality, the results suggest that the intra-articular environment often preserves ACL stumps and facilitates some biological reattachment processes [14].

A 2005 study by Evan H. Crain et al. examined scar formation after ACL tears in 48 patients undergoing reconstruction. They found that 38% had scar tissue in the PCL, 8% had scar tissue extending to the roof of the notch, 12% had ACL remnants adhering to the notch wall or femoral condyle, and 42% had no identifiable ligamentous tissue. Changes in knee laxity were linked to scar formation patterns, with the most significant laxity increase in knees where the ACL adhered to the femur. These results highlight the importance of evaluating ligament adhesion in ACL tear cases during follow-ups [15].

Another finding of our study was that 49 individuals (56.3%) had medial meniscus injuries, 5 individuals (5.7%) had lateral meniscus injuries, and 2 individuals (2.3%) had meniscal root injuries. The highest prevalence of medial meniscus injury was associated with the group without ACL remnant to PCL adhesion (76.9%), while the highest prevalence of lateral meniscus injury was in the group with ACL remnant to PCL adhesion (6.8%), and the highest prevalence of meniscal root injury was in the group with adhesion (2.7%). No significant correlation was found between ACL to PCL adhesion and the presence of meniscal injuries. In a study by Anderson and colleagues, it was shown that the central pivot of the anterior portion of the PCL, in relation to the tibial plateau, was located 1.6 mm from the peripheral edge of the posterior root of the medial meniscus. This finding could help explain the occurrence of a meniscal root tear alongside a PCL tear during the acute phase [16]. In addition, in another study conducted in 2022 aimed at evaluating combined meniscal repair and ACL reconstruction, it was observed that certain patterns of meniscal tears associated with ACL tears, such as root tears and ramp lesions, are not well visualized on MRI compared to complete radial tears or bucket-handle tears. Timely treatment of these tears significantly improves outcomes in ACL reconstruction. Approximately 17% of patients with ACL tears also have lateral meniscus root tears. The mechanisms of stress and increased posterior slope are both associated with the occurrence of lateral meniscus root tears [17].

In 2023, a study was conducted to assess how treatment following acute ACL tears influences secondary meniscal and chondral lesions. The findings indicated that ACL reconstruction does not always prevent osteoarthritis after knee trauma. However, the evidence suggests that ACL reconstruction can prevent secondary joint injuries, such as meniscal lesions, and may improve the success of meniscal repair when performed concurrently with ACL reconstruction. The study highlights a significant risk of lateral meniscus injuries in the soft tissues surrounding the ligament, emphasizing the importance of identifying and addressing these injuries during ACL reconstruction. These meniscal injuries serve as secondary stabilizers, enhancing mobility and providing long-term cartilage protection. Furthermore, in cases of combined ACL injury and meniscal damage that is amenable to repair, ACL reconstruction is recommended [18].

In addition our study results demonstrated that 2.3% of participants had concurrent MCL injuries, and 5 participants (5.7%) had concurrent PCL injuries. The highest number of concurrent MCL injuries was observed in the adhesion group, with 2 participants (2.7%), while the highest number of concurrent PCL injuries again occurred in the adhesion group, with 4 participants (5.4%). However, no significant relationship was observed between ACL adhesion to PCL and concurrent injuries to other cruciate ligaments. In a similar study conducted in 2019, researchers evaluated the progressive changes in the morphological patterns of traumatic ACL tears over time. Distinct patterns of ACL tears were identified, which correlated with the timing of the injury. The first pattern, observed an average of 2.6 months post-injury, appeared as a separate impact without tissue damage. Within 6 months post-injury, two additional patterns emerged: one where the tear adhered to scar tissue and localized within the femoral notch. The final morphological pattern, observed three months after the injury, showed signs of impingement without an evident rupture in the ACL.

This stage progressed into a residual femoral scar, which initially extended to tibial marks and ultimately attached to the posterior cruciate ligaments. The study emphasized the potential for recovery and the biological relevance of these changes over a three-month period [19]. Further results from our study showed that 1 person (1.1%) had chondral lesions, and 18 individuals (20.7%) experienced articular cartilage injuries. The highest incidence of chondral lesions was in the ligamentous adhesion group (1.4%), while the highest percentage of cartilage injuries was in the non-adhesion group (23.1%).

Although more individuals in the adhesion group (15 individuals) had cartilage injuries compared to the non-adhesion group, there was no significant association between ACL remnant-to-PCL adhesions and cartilage injuries. A 2006 study found that the articular cartilage of the patella groove is more vulnerable to damage than the femoral condyle cartilage in cases of ACL injuries, especially in short-duration injuries [20]. In a similar study, an investigation was conducted on ACL tears accompanied by localized defects in the deep knee articular cartilage. It was demonstrated that acute ACL injuries often involve damage to the articular cartilage and subchondral bone beneath the cartilage. These injuries can lead to defects in the deep articular cartilage, causing disability, pain, and presenting a therapeutic challenge in patients with instability and combined pain. In other words, according to the results, all patients with complete ACL tears had defects in the medial femoral condyle articular cartilage [21]. In another study conducted in 2023, the aim was to investigate the concurrent effects of cartilage restoration and ACL surgery on knee injuries in football players. The results indicated that patients with ACL tears are typically affected by cartilage injuries as well [22].

These studies highlight that soft tissue damage, including articular cartilage injury, is common after ACL reconstruction, and proper assessment is essential for effective recovery. In our study, 71.4% had a negative Lachman test, while 28.6% tested positive. The highest percentage of negative results was in the group without ligament attachment (33.2%), and the highest positive Pivot Shift results were in the group with ligament attachment (66.8%). No significant correlation was found between the tests and ligament attachment. A 2022 study emphasized that the Pivot Shift test is the most reliable for diagnosing ACL tears, while the Lachman test's accuracy, particularly for acute cases and complete tears, was suboptimal. Further research is needed to refine the Lachman test's diagnostic algorithms [23].

The effective use of clinical tests like the Lachman and Pivot Shift tests is crucial for accurate follow-up assessments. In our study, 10 individuals (11.5%) experienced varus instability, with the highest number in the ligamentous attachment group (12.2%). However, no significant correlation was found between varus instability and ACL remnant-to-PCL attachment. A 2017 study on knee instability due to ACL deficiency under posterior tibial load found that varus instability and valgus patterns were more pronounced with ACL weakness. Instability in the non-injured knee increased with posterior tibial pressure, approaching the level of the injured knee. Additionally, combined ACL-MCL or ACL-LCL injuries may exaggerate instability and valgus patterns even without significant posterior tibial load [24].

In a study published in 2023, the authors examined the relationship between ACL remnant adhesion to PCL and various clinical outcomes, including Lysholm scores, in patients undergoing ACL reconstruction. The study found no significant association between ACL remnant adhesion to the PCL and clinical outcomes, suggesting that the presence of such adhesions does not adversely affect knee function or recovery post-surgery [25]. Additionally, a 2022 meta-analysis evaluated the clinical outcomes of combined ACL and ALL reconstruction, reporting improved Lysholm scores and a lower rerupture rate compared to isolated ACL reconstruction [26].

Furthermore, a 2023 study assessed the clinical outcomes of primary ACL reconstruction using a six-strand hamstring autograft, utilizing the Lysholm score among other measures [27]. For instance, a study investigated the association between ACL reconstruction and meniscal repair. The study included 49 patients, comprising 35 men and 14 women, with an average age of 29.71 years (ranging from 16 to 54 years) [28].

Another study analyzed the impact of age and gender on ACL injuries. The study reviewed 505 knee MRI images, including 104 females (20.5%) and 401 males (79.5%), with an average age of 34.5 years (ranging from 10 to 85 years). The findings indicated that ACL lesions were reported in 191 cases (37.8%), with no significant gender predominance observed [29].

Furthermore, one research identified male sex, age under 30 years, and a contact injury mechanism as independent risk factors for concomitant major meniscal tears in patients with ACL injuries. The study found that patients with a contact injury mechanism had an approximately 18-fold increased risk for a major lateral meniscus tear compared to those with a non-contact injury [30]. Previous studies have also shown that the presence of ACL remnants in reconstructive surgery can contribute to the improvement of knee recovery and function. For example, a 2017 study examined the value and significance of preserving ACL remnants in various aspects, such as therapeutic effects, remnant classification, biomechanical evaluation, and its correlation with surgical recommendations.

The study concluded that there is insufficient scientific evidence to support the value of preserving the remnant [31]. In another study, the statistical correlation between clinical and functional findings based on the Lysholm scoring system was examined in the success rate of arthroscopic reconstruction surgery. The study demonstrated that the use of standardized questionnaires such as Lysholm can contribute to a more accurate assessment of surgical success and treatment outcomes [25]

In a study conducted by Lee et al. (2018), the authors examined the role of isolated PCL reconstruction in knees with combined PCL and posterolateral complex injuries. The study found that patients who underwent isolated PCL reconstruction experienced significant improvements in knee stability and function. This suggests that addressing ligamentous adhesions to the PCL can lead to favorable clinical outcomes [32].

Furthermore, a review by Wang et al. (2002) discussed the complexities of PCL injuries and their management, highlighting the importance of accurate diagnosis and appropriate surgical intervention to prevent complications such as joint instability and post-traumatic osteoarthritis [32]. Given the existing evidence of molecular interactions associated with ACL injury and its repair, as well as the established links to inflammatory conditions, further investigation into these connections in future studies could greatly enhance our understanding. Exploring these relationships could offer valuable insights, shedding light on the underlying mechanisms and complementing the existing data, thereby contributing to a more comprehensive understanding of ACL injury and its repair process.

## Conclusion

In this study, we investigated the correlation between ACL remnant adhesions to the PCL and knee joint injuries in patients undergoing ACL reconstruction surgery. Our findings revealed that neither age nor gender significantly influenced the development of ACL remnant adhesions. Despite a high prevalence of adhesions, no significant relationship was found between ACL remnant adhesions and chondral lesions, articular cartilage injuries, or meniscal injuries. Furthermore, clinical tests, including the Lachman and Pivot shift tests, showed no significant association with ACL remnant adhesions. These results suggest that while ACL remnant adhesions are common, they do not substantially affect other knee joint injuries or clinical outcomes. Further research is needed to explore the implications of these adhesions on knee health and recovery, with the aim of improving patient management and surgical strategies.

## Conflicts of Interest

The authors declare no conflicts of interest.
